# Improved antitumour immunity in murine neuroblastoma using a combination of IL-2 and IL-12

**DOI:** 10.1038/sj.bjc.6600928

**Published:** 2003-05-13

**Authors:** K E Siapati, S Barker, C Kinnon, A Michalski, R Anderson, P Brickell, A J Thrasher, S L Hart

**Affiliations:** 1 Molecular Immunology Unit, Institute of Child Health, London, UK; 2 Oncology and Haematology Department, Great Ormond Street Hospital for Children NHS Trust, London, UK; 3 Department of Haematology, Royal Free Hospital School of Medicine, London, UK

**Keywords:** neuroblastoma, interleukin-2, interleukin-12, immunotherapy, nonviral vectors

## Abstract

Neuroblastoma immunotherapy using cytokine-modified tumour cells has been tested in clinical trials. However, because of the complex nature of antitumour immune responses, a number of therapies may be required for complete tumour eradication and generation of systemic immunity. We report here the improved antitumour effect of two cytokines, interleukin-2 (IL-2) and interleukin-12 (IL-12), when coexpressed by neuroblastoma cell lines. Initially, transfection of human and mouse neuroblastoma cell lines resulted in high expression levels of biologically active IL-2 and IL-12 *in vitro*. These cytokines when expressed by transfected Neuro-2A cells completely abolished their *in vivo* tumorigenicity in a syngeneic neuroblastoma model. Vaccination of established tumours with IL-12-producing cells exhibited a clear effect with reduced tumour growth in the presence of IL-2. *In vivo* depletion studies showed that CD4^+^ and CD8^+^ T cells mediate the response against cytokine-producing cells. These results suggest that IL-2 and IL-12, when cotransfected in tumour cells, are effective against established disease and provide a promising immunotherapeutic approach for the treatment of neuroblastoma.

Neuroblastoma is one of the most common solid tumours occurring in children. Prognosis of the disease ranges from spontaneous regression for Stage IV S patients to other forms associated with relapse and low survival rates when it occurs as disseminated disease. Neuroblastoma therapy depends on the stage of the disease, but has mainly consisted of chemotherapy followed by surgical resection of the tumour or radiotherapy (Pinkerton *et al*, 2000). Strategies to address the minimal residual disease have included high-dose chemotherapy with autologous haematopoietic stem cell rescue or differentiation of the remaining malignant cells with retinoids (Philip *et al*, 1997). However, the success rate has been limited and thus alternative strategies including immunotherapy are under investigation (Bowman *et al*, 1998).

Inflammatory cytokines such as interleukin-2 (IL-2) and interleukin-12 (IL-12) have been explored for the treatment of cancers (Parmiani *et al*, 2000; Rosenberg, 2001). Interleukin-12 is a potent activator of natural killer (NK) cells and T lymphocytes, and promotes the development of CD4^+^ Th1-inducing antitumour immunity (Trinchieri, 1998). Many of its functions are mediated through the actions of IFN-*γ*, but it has also been shown to synergise with IL-2 to generate lymphokine-activated killer (LAK) cells against tumour targets (Cameron *et al*, 1990). Interleukin-2 has shown antitumour activity in a number of studies mainly because of its ability to activate and enhance the cytolytic activity of NK and T cells (Fearon *et al*, 1990; Gansbacher *et al*, 1990).

Interleukin-2 and -12 have both distinct and overlapping effects on a wide range of immune cell types. They work synergistically on NK cells through the upregulation of IL-12 receptors and STAT4 by IL-2 (Wang *et al*, 2000). Interleukin-12 induces CD25 expression on T cells, thereby enhancing their proliferation in response to IL-2 (Nguyen *et al*, 2000). Natural Killer cells treated with a combination of IL-2 and IL-12 exhibit enhanced *in vitro* cytolytic activity and lyse neuroblastoma cells (Rossi *et al*, 1994) and human osteosarcoma cells more efficiently (Mariani *et al*, 2000). Furthermore, the combination of these two cytokines has proved successful in the treatment of Lewis lung carcinoma (Tanaka *et al*, 2000).

We have previously described LID, a synthetic vector that consists of a lipid (L), an integrin-targeting peptide (I), and a DNA component (D) that transfects a number of cell types with high efficiency (Hart *et al*, 1998). This vector exploits the ubiquitous expression of integrin receptors on the surface of most cell types as a means of binding and entry. A further advantage of this system is that it is not limited by DNA size constraints, as is usually the case with viral systems. The aim of the current study was to use this nonviral vector to deliver genes encoding IL-2, IL-12, or a combination of these cytokine genes, to neuroblastoma cells in an attempt to develop a therapeutic cellular vaccine.

Neuroblastoma cell lines, transfected *ex vivo* with the LID vector, were able to express high levels of IL-2 and IL-12, even if cotransfected with other cytokine genes. These cytokines exhibited *in vitro* biological activity and reduced the tumorigenicity of neuroblastoma cells in a mouse model. Also, the combination of IL-2 and IL-12, when expressed by murine neuroblastoma cells, was capable of eradicating established tumours and exhibited a greater antitumour effect than either cytokine alone.

## MATERIALS AND METHODS

### Cell culture

The human neuroblastoma cell lines SHSY-5Y, IMR-32, and the mouse cell line Neuro-2A (N2A), derived from a spontaneous neuroblastoma tumour in A/J mice, were obtained from the American Tissue Culture Collection (ATCC) or the European Collection of Cell Cultures (ECACC). They were cultured in Dulbecco's modified Eagle medium (DMEM-Glutamax-1) (Life Technologies, Paisley, UK) supplemented with 10% foetal bovine serum (Sigma Chemical Co, Dorset, UK), 1% nonessential amino acids, 100 IU ml^−1^ penicillin and 100 *μ*g ml^−1^ streptomycin, and 1% sodium pyruvate (all from Life Technologies, Paisley, UK). The hybridoma cell lines YTS191.1 and YTS169 were a gift from Dr Baker (Institute of Neurology, London, UK) and produce rat (IgG_2b_) anti-mouse CD4 and CD8 antibodies, respectively. Cells were cultured in RPMI containing 10% FCS, 2 mM glutamine, and 10 *μ*g ml^−1^ gentamicin until confluent. The medium was then replaced with Hybridoma-SFM medium (Life Technologies, Paisley, UK) containing 10 *μ*g ml^−1^ gentamicin for 48 h.

### Plasmid DNA

The pEGFP-N1 plasmid encodes the gene for the green fluorescence protein and was obtained from Clontech (CA, USA). The plasmid pCMV-Flexi12 encodes the cDNA for both human IL-12 genes (p35 and p40) as a fusion protein (Anderson *et al*, 1997). pCI-IL2 plasmid carrying the human IL-2 cDNA was constructed by incorporation of the hIL2 cDNA into the *EcoR*I–*Not*I restriction sites of the pCI plasmid (Promega, Southampton, UK). The cDNA for both genes of murine IL-12 were carried on the expression vector pCDNA3.1scIL12 with a linker and expressed as a fusion protein [a gift from Professor Reisfeld (The Scripps Research Institute, CA, USA)] (Lode *et al*, 1998). All plasmids were propagated in *E. coli* DH5*α* and purified on Hybaid columns according to the manufacturer's instructions (Hybaid, Ashford, UK).

### Optimisation of neuroblastoma cell line transfection with the LID vector

Cells were seeded in a 48-well plate at a concentration of 2.5 × 10^4^ cells per well and transfected using the LID vector, as described previously, using 1 *μ*g of DNA per well (Hart *et al*, 1998). LD complexes were prepared by mixing Lipofectin with plasmid DNA at a weight ratio of 5 : 1. All complexes were incubated for 1.5 h at RT prior to transfecting the cells for 4 h. Previous studies (Hart *et al*, 1998) showed that all the three vector components were required for maximum transfection efficiency. Transfections of neuroblastoma cells with the full LID formulation were significantly more efficient than those with LD or ID complexes (*P*<0.05) (data not shown). The most consistent transfection efficiency was achieved by an *α*5*β*1 integrin-specific peptide (peptide 6), whose binding domain RRETAWA was isolated from a phage display library (Koivunen *et al*, 1994).

### FACS analysis

Transfection efficiency of neuroblastoma cells with pEGFP-N1 plasmid vector was estimated through fluorescence-activated cell sorting (FACS) analysis. The cells were transfected as described above in 48-well plates using the pEGFP-N1 vector and analysed 48 h post-transfection using a FACS sorter (FACSCalibur, Beckton Dickinson, Oxford, UK) and Cell Quest software.

### Analysis of cytokine production

Transfection of neuroblastoma cells with the cytokine expression vectors was carried out in 24-well plates using peptide 6. The supernatant from transfected cell lines was collected at 24 h intervals and quantitation of IL-2 or IL-12 was performed using a DuoSet ELISA Development System (R&D Systems, Oxford, UK) according to the manufacturer's instructions.

### Cell proliferation assays

Mononuclear cells from the peripheral blood of healthy donors were obtained by density gradient centrifugation on Ficoll-Paque (Amersham Pharmacia Biotech, St Albans, UK). These cells were monocyte-depleted by plastic adherence and then resuspended at 10^7^ cells ml^−1^ in RPMI 1640 medium (Life Technologies, Paisley, UK) containing 2.5 *μ*g ml^−1^ PHA (Sigma Chemical Co, Dorset, UK) and cultured for 3 days as described previously (Anderson *et al*, 1997). rhIL-2 (50 IU ml^−1^) (R&D Systems, Oxford, UK) was added for a further 24 h and 50 *μ*l of cell suspension (4 × 10^5^ cells ml^−1^) was incubated with the supernatant of transfected neuroblastoma cells. Cell proliferation was measured after incubation with ^3^H-labelled thymidine (0.5 *μ*Ci per well) for 16 h.

### Animal studies

All animal procedures were approved and licensed by the Home Office and performed according to the standards required by the UKCCCR (Workman *et al*, 1998). A/J mice were obtained from Harlan Laboratories (UK). *γ*_c_/RAG2 knockout mice were a gift from JP Di Santo (Pasteur Institute, Paris, France) (Goldman *et al*, 1998). A/J (*n*=6 per group) or *γ*_c_/RAG2 knockout mice (*n*=3 per group) were inoculated subcutaneously in the posterior flank with 10^6^ N2A cells transfected with LID vector complexes containing cytokine genes or control plasmid, or were untransfected. For vaccination of established tumours, tumour formation was induced by subcutaneous inoculation of 10^6^ wild-type N2A cells. Palpation was performed to examine tumour size, which reached a diameter of ∼5 mm at 10±1 days post-inoculation. Subcutaneous tumour growth was monitored using callipers by measuring their diameter every 2–3 days. Animals were killed when tumours reached a diameter of >17 mm, which was defined as the survival end point. *In vivo* T- and NK-cell immunodepletion experiments were performed by intraperitoneal administration of 350 *μ*g rat IgG_2a_ anti-mouse CD4^+^, anti-CD8^+^, or 40 *μ*l rabbit anti-asialo GM (Wako, Neuss, Germany), respectively, into A/J mice on days −6, −3, +2, and every 5 days thereafter. The anti-CD4^+^ and anti-CD8^+^ antibodies were purified from the culture supernatant of YTS191.1 and YTS169 hybridoma cells on HiTrap Protein G columns (Amersham Pharmacia Biotech, St Albans, UK) according to the manufacturer's recommendations and dialysed with PBS. On the day of tumour inoculation, the splenocytes of one animal per group were stained for CD4, CD8, and/or NK cells, in order to confirm immunodepletion using rat FITC-conjugated anti-mouse CD4, anti-CD8, and pan-NK or NK 1.1 antibodies (Pharmingen, San Diego, CA, USA). Flow cytometric analysis was performed as described above.

### Statistical analysis

The nonparametric Mann–Whitney test was used to assess the significance between different experimental groups. For cytokine production and bioassays, comparison among different groups was performed at each time point.

## RESULTS

### Cytokine expression by neuroblastoma cell lines

LID transfection was optimised for the human and mouse neuroblastoma cell lines. In all cases, the most efficient vector formulation consisted of an *α*_5_*β*_1_ integrin-targeting peptide and lipofectin, as described in the Materials and Methods. Transfection of neuroblastoma cell lines with the plasmid pEGFP-N1 encoding the green fluorescent protein (GFP) reporter gene, using the LID vector, was highly efficient and ranged from 47% for SHSY5Y to 64% for N2A and IMR-32 cells.

**Figure 1:**
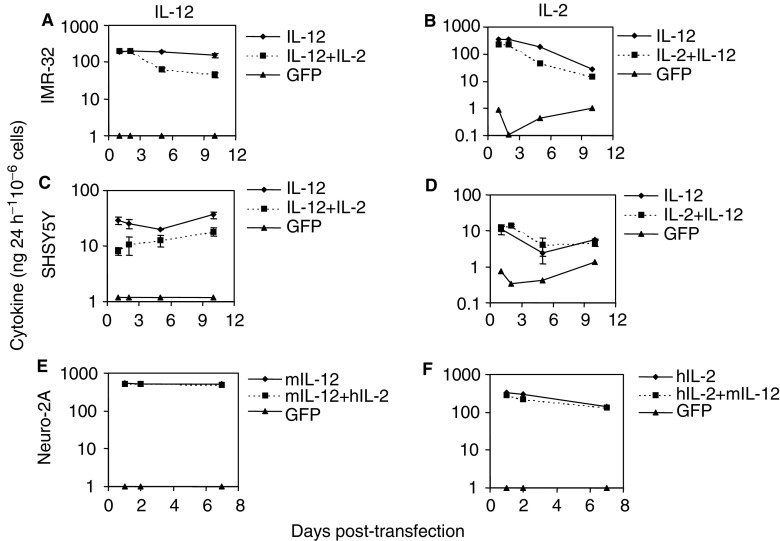
*In vitro* IL-2 and IL-12 expression by LID-transfected neuroblastoma cell lines. Neuroblastoma cells were transfected using an *α*_5_*β*_1_-specific peptide and pCI-IL2, pCMVFlexi-12, or pCDNA3.1scIL12 plasmids. Cytokine expression was monitored over a period of 10 days by collecting the cell culture supernatant at 24 h intervals and assaying the cytokine levels by ELISA. Human IL-2 (**A**) and IL-12 (**B**) secretion by transfected IMR-32 cells; (**C**), (**D**) SHSY5Y cell line; (**E**), (**F**) hIL-2 and murine IL-12 expression by transfected N2A cells, respectively. Solid lines represent transfections with a single cytokine gene, and dotted lines represent experiments where both cytokine genes were introduced into the cells. Error bars represent standard deviations from triplicate transfections and one representative experiment is shown.

Cytokine expression was studied after transfecting human neuroblastoma cell lines IMR-32 and SHSY5Y with pCI-IL2 vector and/or pCMV-Flexi12 (human) (Anderson *et al*, 1997), while the mouse Neuro-2A cell line was transfected with pCDNA3.1scIL12 (mouse) (Lode *et al*, 1998) and/or pCI-IL-2. For both transfected IMR-32 and SHSY5Y cell lines, IL-12 expression followed a stable pattern with a slight reduction in expression on day 5 in the latter cell line. Transfection experiments using single cytokine genes resulted in significantly higher production of IL-12 than in experiments where IL-2 and IL-12 genes were cotransfected (*P*<0.05), despite the fact that equal amounts of each cDNA were used to transfect these cells. Nevertheless, hIL-12 levels in double transfections were sustained above 45 ng 24 h^−1^ 10^−6^ cells in IMR-32 (Figure 1A

) and 10 ng 24 h^−1^ 10^−6^ cells in SHSY5Y cell line (Figure 1C) throughout the 10-day period. Murine IL-12 expression by mouse N2A cells was sustained at a fairly constant level throughout the seven days of analysis with no significant difference between single (530±6 ng 24 h^−1^ 10^−6^ cells) and cotransfection experiments (513.6±17 ng 24 h^−1^ 10^−6^ cells) (*P*>0.05) (Figure 1E).

Human IL-2 secretion by human neuroblastoma cell lines reached a peak 24–48 h post-transfection in both cell lines, with IMR-32 cells expressing 349.7±5.5 ng 24 h^−1^ 10^−6^ cells (Figure 1B) and SHSY5Y cells 14.3±1.25 ng 24 h^−1^ 10^−6^ cells (Figure 1D). There was a slight decrease in hIL-2 levels after day 5 but expression was maintained above 5 ng 24 h^−1^ 10^−6^ cells at least up to 10 days of culture. The levels of hIL-2 secreted by N2A cells were higher, starting at 385 ng 24 h^−1^ 10^−6^ cells and dropping to 170 ng 24 h^−1^ 10^−6^ cells by day 7 of the analysis (Figure 1F). In all experiments, there was minimal detectable production of cytokines by pEGFP-N1-transfected cells (*P*<0.04).

**Figure 2:**
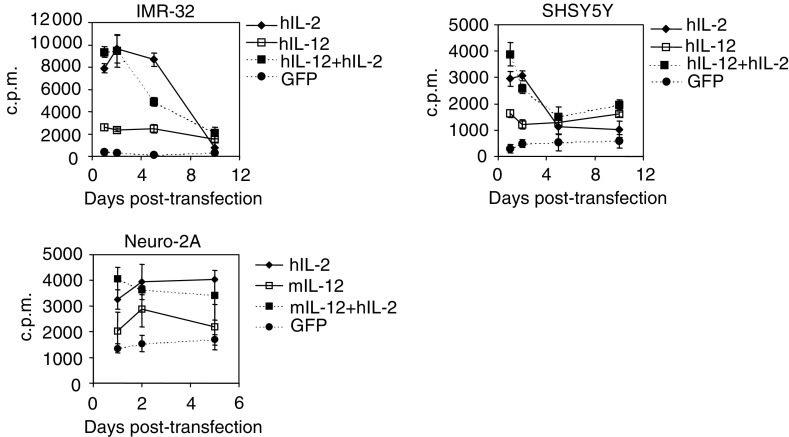
Biological activity of cytokines secreted by transfected neuroblastoma cell lines. The bioactivity of IL-2 and IL-12 expressed by neuroblastoma cell lines was tested on human lymphoblasts after stimulation with PHA and rhIL-2. Cell proliferation was assessed by ^3^H-labelled thymidine incorporation over a 16-h incubation time. Means±s.d. were calculated from triplicates. Results are expressed as scintillation counts per minute (c.p.m.) and one representative experiment is shown.

The biological activity of IL-2 and IL-12 produced by the neuroblastoma cells was tested on PHA-stimulated peripheral blood lymphocytes. Undiluted supernatant from cytokine-transfected cells was applied onto PHA-activated lymphocytes for 24 h prior to ^3^H-labelled thymidine addition. The proliferative effect of the cytokines on PHA-activated lymphocytes after incubation with ^3^H-labelled thymidine for 16 h was examined. Biologically active IL-2 was secreted by transfected SHSY5Y and IMR-32 cells up to 5 days post-transfection (*P*<0.05), although there was a decline in the H^3^-labelled thymidine incorporation into PHA-stimulated lymphocytes after day 2 (Figure 2

). IL-2 expressed by transfected Neuro-2A cells exhibited an increasing proliferative effect on activated lymphocytes, which reached a plateau by day 5 and was significantly higher than supernatant from control transfectants (*P*<0.05). Human IL-12 secreted by transfected SHSY5Y and IMR-32 cells over a period of at least 10 days *in vitro* showed a stable level of biological activity compared to control supernatant (*P*<0.05). From 48 h after transfection of Neuro-2A cells with murine IL-12, cytokine levels in the supernatant were sufficient to induce a significant proliferative effect on PHA-activated lymphocytes, which persisted throughout the 5-day analysis period (*P*<0.06). Furthermore, the supernatant from all neuroblastoma cell lines transfected with genes for both IL-2 and IL-12 exhibited a significant mitogenic effect compared to control supernatant (*P*<0.05), indicating that cotransfection results in expression of biologically active cytokines for at least 10 days *in vitro*.

### *In vivo* tumourigenicity of cytokine-transfected neuroblastoma cells

**Figure 3:**
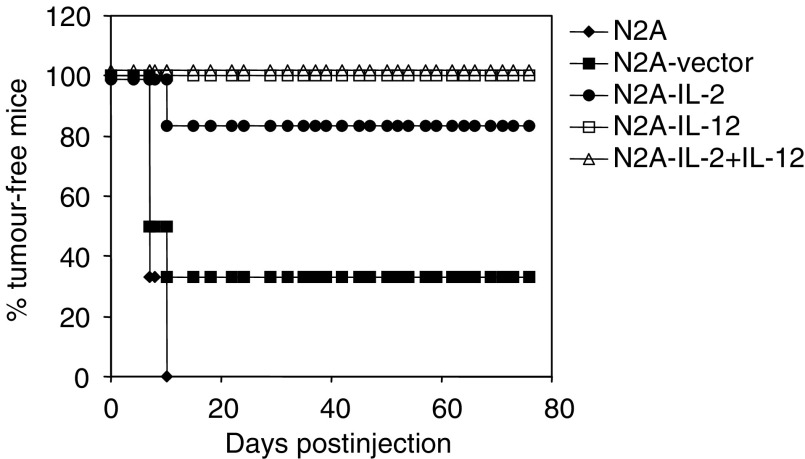
*In vivo* tumorigenicity of cytokine-transfected versus untransfected N2A cells in syngeneic mice. In total, 10^6^ untransfected, vector control, IL-2-, IL-12- or IL-2- and IL-12-producing cells were injected subcutaneously into A/J mice (*n*=6 per group). Data from one representative experiment out of two are shown.

To establish whether the cytokines produced by neuroblastoma cells were biologically active *in vivo* and to test the vaccine in the A/J mouse model of neuroblastoma, 6–8-week-old mice were inoculated subcutaneously with 10^6^ unmodified or cytokine-transfected N2A cells. Mice receiving cells transfected with IL-12 or a combination of IL-2 and IL-12 remained tumour free for at least 75 days. Interleukin-2-secreting N2A cells also failed to grow tumours in 83% of the animals compared to mice that received untransfected cells that developed tumours within 12 days of the injection (Figure 3

). The tumorigenicity of cytokine-transfected cells in A/J mice was reduced significantly (*P*<0.05) compared to cells transfected with the control plasmid. However, 33% of animals inoculated with cells transfected with plasmid control remained tumour free. We determined whether transfection was altering the proliferative potential of the cells by ^3^H-thymidine incorporation. No differences were observed among untransfected, cytokine- or vector-transfected N2A cells, suggesting that transfection itself did not influence the cells' *in vivo* tumour-forming potential (data not shown).

**Figure 4:**
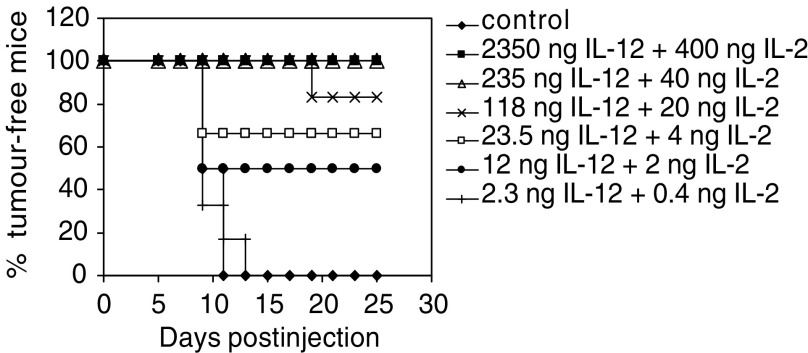
*In vivo* titration of the cytokine levels required for an antitumour effect. N2A-IL-2+IL-12 (10^3^–10^6^) were coinjected with 10^6^ wild-type N2A cells into A/J mice (*n*=6 per group). Tumour formation was monitored over 30 days and the survival of animals in each group from one representative experiment is plotted over time.

The requirement for secreted cytokines for an antitumour effect was determined by performing tumourigenicity experiments in which groups of A/J mice (*n*=6) were coinjected with viable N2A cells (10^6^) and with a dilution series (10^3^–10^6^ cells) of N2A-IL-2+IL-12 cells. The control group consisted of animals receiving only wild-type N2A cells (10^6^). A complete antitumour effect was observed with N2A-IL-2+IL-12 expressing 235 ng IL-12 and 40 ng IL-2 (Figure 4

). However, efficient antitumour protection (83%) were achieved with 118 ng IL-12 and 20 ng IL-2, while for 50% tumour growth inhibition a minimal 12 ng IL-12 and 2 ng IL-2 was required.

### Eradication of established tumours

**Figure 5:**
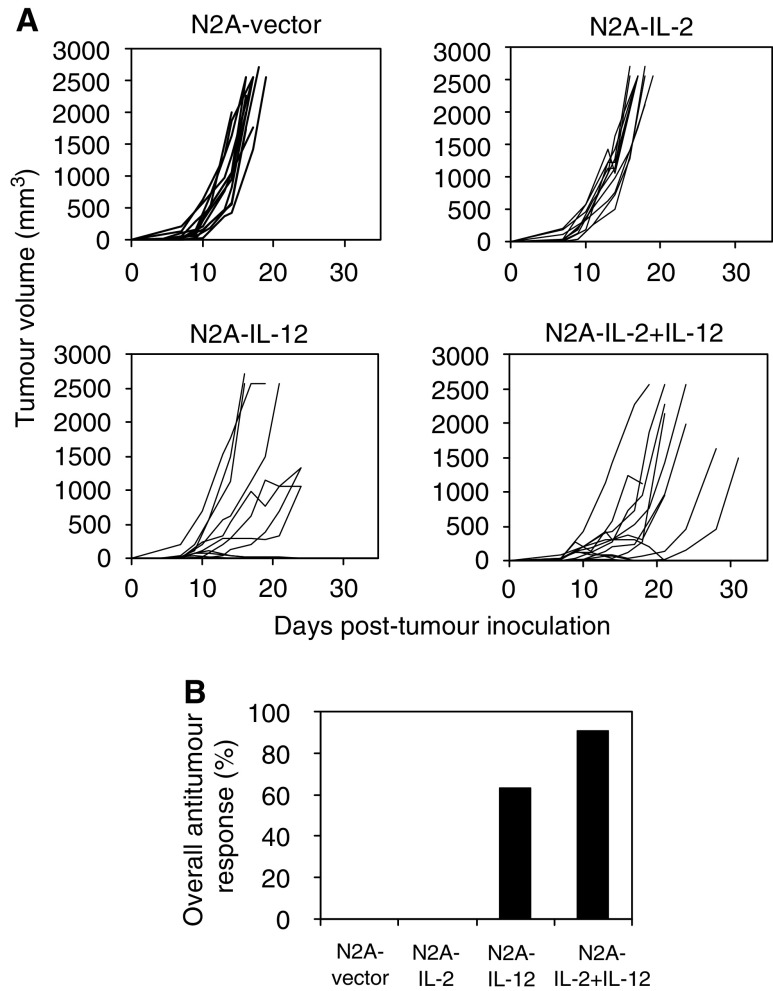
Effect of vaccination on established murine tumours. A/J mice (*n*=11–12 per group) were subcutaneously inoculated with 10^6^ parental N2A cells and 9–11 days later vaccinated directly into the tumour with an equal number of irradiated (25 Gy) vector- or cytokine-transfected N2A cells. The tumour growth in each animal was monitored and is represented by a single line (**A**). The overall antitumour response combining tumour growth inhibition and tumour eradication are also shown (**B**). Data from two separate experiments were combined.

The effect of cytokine-transfected N2A cells on established tumours was examined by inducing subcutaneous neuroblastoma tumours in A/J mice by inoculation of 10^6^ wild-type N2A cells. When a minimal tumour was palpable (approximately 5 mm diameter), the animals received an intratumoral vaccination with 10^6^ vector or cytokine-transfected N2A cells that had been previously irradiated (25 Gy). Irradiation of cells reduced their *in vitro* proliferation, but did not significantly alter their cytokine expression levels compared to nonirradiated transfected N2A cells (data not shown). Tumour growth in vaccinated animals was monitored every 2 days. The tumours of mice vaccinated with vector-transfected or IL-2-transfected N2A cells rapidly reached a diameter of >17 mm and had to be killed, 16.8±1.4 days and 17.3±0.9 days post-vaccination, respectively (Figure 5A

). There was significant survival enhancement when vaccination was performed with N2A-IL-2+IL-12 (25.3±7 days; *P*<0.03) or N2A-IL-12 cells (31±17 days; *P*<0.05) compared to N2A-vector cells.

Interestingly, the N2A-IL-12 and the N2A-IL-2+IL-12 vaccines exhibited similar levels of efficiency of eradication (27 and 33%, respectively) on established tumours. However, in addition to mice displaying tumour eradication, a further 58% of N2A-IL-2+IL-12-vaccinated animals responded to vaccination by tumour growth inhibition. In contrast, only 36% of animals, that received the N2A-IL-12 vaccine, showed tumour growth delay despite higher IL-12 expression levels, while the tumours in the remaining animals (37%) grew aggressively. This indicated that vaccination with N2A-IL-2+IL-12 cells generated a superior overall antitumour effect with 91% of treated animals displaying tumour eradication or retarded growth, compared with cells expressing IL-12 alone, where only 63% of treated animals showed one of these responses (Figure 5B).

### Involvement of CD4 and CD8 T cells in the initial immune response against tumour cells expressing IL-2 and IL-12

**Figure 6:**
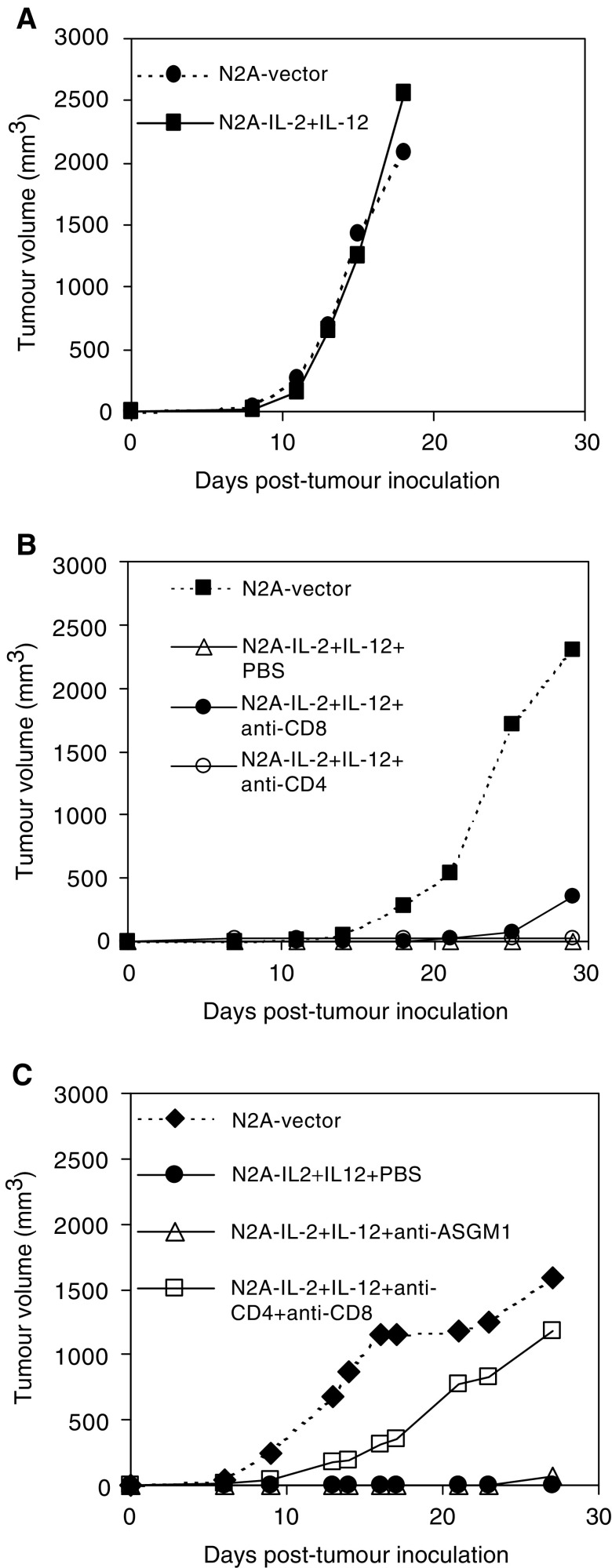
Tumorigenicity of N2A-IL-2+IL-12 transfectants in *γ*_c_/RAG2 knockout (**A**) or in immunodepleted (**B**, **C**) mice. Administration of anti-CD4 or anti-CD8 antibodies was performed separately (**B**) or coadministered (**C**) intraperitoneally twice before inoculation of N2A-IL-2+IL-12 cells and every 5 days thereafter. Natural Killer cell depletion was performed by i.p. Inoculation of anti-ASGM1 antibody and control group consisted of PBS-injected animals. Tumour growth of N2A-IL-2+IL-12 cells in immunodepleted mice was compared with mock-transfected N2A-cells (N2A-vector). The mean tumour volume of six mice per group is presented.

The mechanism of cytokine-mediated tumour cell rejection was assessed in tumourigenicity studies performed in *γ*_c_/RAG2 knockout mice, which lack any T, B, or NK cells (Goldman *et al*, 1998). Subcutaneous administration of N2A-IL-2+IL-12 transfectants into *γ*_c_/RAG2 knockout mice showed tumour growth kinetics similar to those of N2A-vector cells (Figure 6A

). This indicated that abrogation of tumourigenicity of N2A-IL-2+IL-12 cells was an immunological phenomenon. To further specify the effector population responsible for the rejection of N2A-IL-2+IL-12 cells, we performed immunodepletion studies in A/J mice. Mice (*n*=6) were depleted of either CD4^+^, CD8^+^, CD4^+^ and CD8^+^, or NK cell subsets as described in Materials and Methods or received PBS as a control. Prior to inoculation of N2A-IL-2+IL-12 cells, one animal per group was killed to confirm the extent of immunodepletion (data not shown). Depletion of CD8^+^ T cells did not have an effect on the tumourigenicity of N2A-IL-2+IL-12 cells until day 25 when 33% of the immunodepleted animals grew tumours, while mice receiving PBS as a control remained tumour free. This indicated that these effector cells were at least partly involved in the rejection of N2A-IL-2+IL-12 transfectants (Figure 6B). Depletion of NK cells in A/J mice with anti-asialo GM antibody did not induce tumour formation with viable N2A-IL-2+IL-12 cells, whereas depletion of both CD4^+^ and CD8^+^ T-cell populations resulted in aggressive tumour formation in all animals (Figure 6C). Interestingly, the kinetics of tumour formation by N2A-IL-2+IL-12 cells in CD4^+^- and CD8^+^-depleted mice showed a slight time delay compared to those of N2A-vector cells, suggesting that depletion is incomplete or that NK cells are active.

## DISCUSSION

Evasion of the host immune surveillance may contribute to tumour development. A number of mechanisms have been proposed to explain the failure of the immune system to attack tumour growth including downregulation or absence of MHC, costimulatory molecules or other antigen-processing machinery, secretion of inhibitory cytokines, or loss of immunodominant antigens. Whatever the mechanism, immunotherapy has emerged as a promising approach for the eradication of parental tumours and generation of long-term immunity (Foss, 2002; Paul *et al*, 2002). The processes of tumour development and eradication are complex and still poorly understood. Different cytokines affect the immune response in different ways and therapeutic efficacy depends on a number of steps from the initial tumour-specific immune activation to the development of long-lasting protective immunity. In many cases, combinations of cytokines or coexpression of MHC or costimulatory molecules have proved more efficient in recruiting inflammatory cells to the tumour site, resulting in the eradication of established animal tumours (Putzer *et al*, 1997; Addison *et al*, 1998; Chong *et al*, 1998).

Current therapy for neuroblastoma consists of surgical resection, radiation, or chemotherapy with a mean disease-free survival of 3 years (Philip *et al*, 1997; Pinkerton *et al*, 2000). However, because of the high incidence of relapse, more effective treatments targeting minimal residual disease and metastases formation are required. This was addressed in a study with an anti-GD2 antibody fused to murine IL-12 (Lode *et al*, 1998). Also, patient clinical trials using IL-2 gene-modified autologous tumour cells have shown antitumour responses with antibody production and MHC-restricted cytotoxicity against autologous tumour cells (Bowman *et al*, 1998).

The use of a combination of IL-2 and IL-12 is supported by a number of studies indicating that cancer eradication is a complex process and might require recruitment of multiple cytokines or costimulatory molecules. Peripheral blood mononuclear cells from neuroblastoma patients showed increased cytotoxic activity when activated *in vitro* with both cytokines than with IL-2 or IL-12 alone (Rossi *et al*, 1994). Furthermore, a synergistic combination between IL-2 and IL-12 was observed in a Lewis lung carcinoma model where only vaccination with dual-gene transduced cells inhibited growth of established tumours (Tanaka *et al*, 2000). In the current study using a nonviral vector system, cytokine expression by transfected neuroblastoma cell lines peaked around 48 h after transfection. Codelivery of two cytokine genes carried on different plasmids resulted in the production of biologically active cytokines. Also, the cytokine levels secreted by transfected neuroblastoma cell lines were comparable to levels reported for adenoviral vectors (Leimig *et al*, 1996). For the purpose of a tumour vaccine, such cytokine levels were shown to be adequate for immunomodulation despite the transient nature of transfection (Balicki *et al*, 2000).

The IL-2 and IL-12 cytokines secreted by transfected N2A cells were not only capable of inducing *in vitro* proliferation of PHA-activated lymphocytes, but also exhibited biological activity *in vivo*. Following inoculation into syngeneic A/J mice, N2A cells transfected with cytokines (mIL-12, hIL-2, or a combination of the two) exhibited significantly reduced tumorigenicity compared to untransfected or vector control-transfected cells, which formed aggressive tumours and had to be killed by 12 days. This effect was not because of reduced cell proliferation as shown by *in vitro* ^3^H-labelled thymidine incorporation, but was rather a specific effect of cytokine expression. *In vivo* the cytokine levels required for complete tumour growth inhibition by parental N2A cells were 235 ng/24 h IL-12 and 40 ng/24 h IL-2. Significant antitumour protection was, however, achieved with N2A-IL-2+IL-12 cells expressing 118 ng IL-12 and 20 ng IL-2.

Studies in *γ*_c_/RAG2 knockout mice indicated that rejection of N2A cells expressing a combination of IL-2 and IL-12 was an immunological phenomenon. Natural Killer depletion in A/J mice had no effect on tumour formation by N2A-IL-2+IL-12 cells, suggesting that this effector cell population is not involved in the rejection of N2A-IL-2+IL-12 cells. Further *in vivo* depletion studies in A/J mice showed that rejection of N2A-IL-2+IL-12 transfectants was mediated partly by CD8^+^ T cells. However, depletion of both T cell subsets completely abrogated the immunogenicity of N2A-IL-2+IL-12 cells, suggesting a tumour rejection mechanism involving CD8^+^ T-cell activation by CD4^+^ T cells. Most cancer vaccines will be used on patients with primary tumours or on those who, after initial treatment, have relapsed with metastatic disease. Therefore, the most clinically relevant aspect of a cancer vaccine is its efficacy on established disease. We examined the immunological effect of irradiated N2A cells expressing IL-2, IL-12, or a combination of the two cytokines on parental tumours. The N2A-IL-2+IL-12 vaccine exhibited a higher overall antitumour response compared to either cytokine used alone. It was effective in most treated animals inducing either tumour regression (33%) or delay in tumour growth (58%), with an overall response rate of 91%. When IL-12 was used on its own, it resulted in similar tumour regression with the N2A-IL-2+IL-12 vaccine with 27% of the tumours undergoing complete regression and an overall response of just 63%, while 37% of animals receiving the IL-12 vaccine grew aggressively. Interestingly, IL-2 alone did not exhibit any antitumour efficacy, despite the fact that it totally abrogated the *in vivo* tumorigenicity of IL-2-transfected N2A cells. Although we were not able to determine the reason for the absence of tumour growth by N2A-vector cells in two out of six animals, vector-related rejection events have been reported previously (Todryk *et al*, 1999).

Although only local vaccine administration was addressed in this study, the biological efficacy of the N2A-IL-2+IL-12 vaccine and its ability to be used against established disease was verified. Further optimisation would be required and alternative vaccine administration routes would have to be tested, however, before such a treatment is considered for clinical use. In addition, it should be emphasised that the antitumour effects of the N2A-IL-2+IL-12 vaccine were a result of single vaccination experiments. The observation that the majority of N2A-IL-2+IL-12-vaccinated tumours exhibited a delay in tumour growth suggests that repeated vaccination is probably required for complete regression.

The improved antitumour effect shown by the combination of IL-2 and IL-12 suggests that an inflammatory cytokine, such as IL-12 functions to attract effector cells at the vaccination site and activate APCs. Its antitumour immunity has also been shown to include non-T-cell-mediated events (Nastala *et al*, 1994) and angiostatic effects (Voest *et al*, 1995). Histological examination of vaccinated tumours revealed infiltration of CD45^+^ cells compared to N2A-vector-treated animals (data not shown). A second cytokine, such as IL-2, that is capable of clonally expanding activated T cells, may be vital for strengthening the immune response against the tumour and achieving long-term immunity. Indeed, tumour-targeted IL-2 has been shown to be capable of amplifying CD8^+^ T cells after IL-12 vaccination and enhancing MHC I-mediated killing in a murine model of neuroblastoma metastasis (Lode *et al*, 1999). Despite the reported synergy between IL-2 and IL-12 on NK cells (Wang *et al*, 2000), immunodepletion studies in A/J mice suggested that NK cells did not play a major role in the rejection of IL-2+IL-12-transfected N2A cells. Consistent with our findings, IL-2 and IL-12 may work together on T-cell activation (Nguyen *et al*, 2000). The complete abrogation of immunogenicity of N2A-IL-2+IL-12 cells, when both T-cell subsets are depleted, supports the latter hypothesis.

In conclusion, in this study, we have shown that coexpression of IL-2 and IL-12 offers improved antitumour immunity in a murine model of neuroblastoma and provides a potential approach for the treatment of neuroblastoma. Also, the proposal that multimodality therapies against cancer may prove more efficient is supported by these findings.
